# Enzymatic Synthesis of Aroma Ester Catalyzed by Lipases Immobilized on Activated Carbon Derived from Fermented Cocoa Bean Shells

**DOI:** 10.1021/acsomega.5c07104

**Published:** 2025-11-06

**Authors:** Márcia Soares Gonçalves, Igor Carvalho Fontes Sampaio, Polyany Cabral Oliveira, Helen Luiza Brandão Silva Ambrosio, Isabela Viana Lopes de Moura, Taíse Amorim Ribeiro, Sabryna Couto Araujo, Paulo Neilson Marques dos Anjos, Cristiane Martins Veloso, Adriano Aguiar Mendes, Muhammad Irfan, Julieta Rangel De Oliveira, Marcelo Franco

**Affiliations:** † Biotransformation and Organic Biocatalysis Research Group, Department of Exact Sciences, 74361 Santa Cruz State University, Ilhéus 45654-370, Brazil; ‡ Institute of Health Sciences, Federal University of Bahia, Salvador 40110-100, Brazil; § Laboratory of Research and Innovation of Advanced Materials, Department of Exact Sciences, 74361 Santa Cruz State University, Ilhéus 45654-370, Brazil; ∥ Process Engineering Laboratory, State University of Southwest Bahia, BR 415, km 04, s/n, Itapetinga 45700-000, Brazil; ⊥ Institute of Chemistry, Federal University of Alfenas, Alfenas 37130-001, Brazil; # Department of Biotechnology, Faculty of Science, 66906 University of Sargodha, Sargodha 40100, Pakistan

## Abstract

This study explored the production of activated carbon from cocoa bean shells (CBS), both before and after solid-state fermentation, and evaluated its potential as a support for enzyme immobilization in aroma ester synthesis. Fermentation improved the physicochemical properties of the shells, increasing surface area and porosity of the resulting activated carbon. Activated carbon from fermented shells (AC-FCBS) displayed a surface area of 1158 m^2^/g, compared to 1050.30 m^2^/g for nonfermented shells (AC-CBS). These supports were tested for immobilization of Amano Lipase PS (APS), achieving relative activity yields of 72% ± 0.11 for AC-CBS, 82% ± 0.16 for AC-FCBS, and 85% ± 0.20 for the commercial resin Diaion HP-20 (DIR). In esterification reactions, APS immobilized on AC-FCBS reached 85% yield, surpassing the 75% obtained with AC-CBS. Regarding operational stability, APS-AC-FCBS retained 95% of its initial activity after five cycles and showed only a 5% reduction in conversion after seven cycles, whereas APS-DIR lost 30% under the same conditions. Notably, competitive results were achieved with lower enzyme concentrations (3 mg/g of support), highlighting the efficiency of the developed material. Overall, AC-FCBS proved to be a promising, low-cost, and effective support that enhances enzyme performance, valorizes agro-industrial residues, and contributes to sustainable esterification processes. These outcomes align with the United Nations Sustainable Development Goal 12 (Responsible Consumption and Production), promoting resource efficiency, waste reduction, recycling, and circular economy practices.

## Graphical abstract

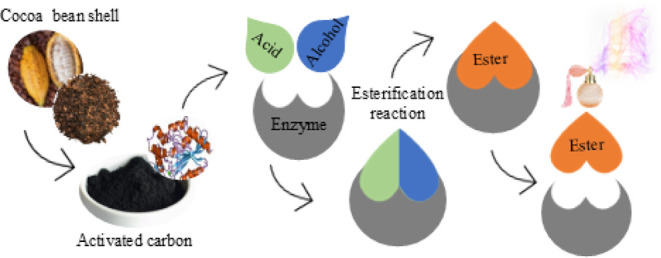

## Introduction

Aromas play a crucial role in directly influencing consumer preferences when purchasing food products. The market for these compounds has expanded significantly, reaching an estimated value of thirty-five point three billion dollars in 2024, with projections indicating further growth to forty-two point three billion dollars by 2029, representing an annual increase of 3.7.^1^ Given their widespread use, understanding efficient and sustainable methods for aroma production is critical for both industrial and consumer applications.

A wide range of food products incorporate flavoring agents, including sauces,^2^ syrups, and soft drinks,^3^ as well as bread, cakes, and cookies.^4^ Additionally, puddings, pies, and creams^1^ are commonly flavored. Floral aromas are widely used in wines,^5^ while fruity aromas are added to vinegars,^6^ cheeses, and yogurts.^7^ Sweet and bitter aromas are applied in cookies, fine chocolates, and bakery products,^8^ whereas citrus aromas are incorporated into soft drinks, juices, and candies.^9^ Beyond the food industry, aromas are extensively applied in cosmetics^10,11^ and pharmaceuticals.^12^

Citrus aromas are predominantly obtained through chemical synthesis via transesterification or direct esterification reactions, utilizing carboxylic acids and short-chain alcohols as substrates^13^ However, these catalytic processes frequently generate toxic and corrosive byproducts, such as hydrochloric and sulfuric acids, require prolonged reaction times and high temperatures, and involve multiple purification steps. Such limitations underscore the need for alternative methods that are both environmentally friendly and economically viable.^14,15^

In this context, enzymatic catalysis has emerged as a promising approach, enabling the production of eco-friendly aromas under mild conditions, with high specificity and stability. Lipases are particularly advantageous due to their catalytic efficiency at low temperatures and pressures. Despite these benefits, the industrial use of free lipases remains limited by high costs, low thermal and chemical stability, and challenges in enzyme recovery and reuse, which increase operational costs and reduce process efficiency.^15^

To address these limitations, enzyme immobilization on solid supports, such as activated carbon (AC) derived from lignocellulosic byproducts, has been widely explored.^16^ Previous studies on AC produced from lignocellulosic residues, such as peach palm heart, tamarind seeds, or coconut shells, highlighted limitations including reduced pore diameter and volume, decreased performance over multiple cycles, and limited effectiveness under high organic contaminant loads.^17^ These constraints reduce the feasibility of large-scale applications and underscore the need for activated carbons with enhanced adsorption capacity and stability.^14^

Bioprocessing techniques, particularly solid-state fermentation (SSF) with filamentous fungi, have demonstrated the potential to overcome these limitations. During SSF, microbial activity selectively degrades lignin, partially depolymerizes hemicellulose, and exposes cellulose, resulting in a more open and accessible matrix.^18^ Additionally, the removal or transformation of noncarbon components prevents pore blockage and promotes a more homogeneous carbon structure. Consequently, fermented biomass yields activated carbon with improved textural and chemical properties, higher enzyme immobilization efficiency, and superior catalytic performance compared to nonfermented precursors.^14,19^

Based on these observations, this study hypothesizes that the strategic bioprocessing of cocoa bean shells prior to activated carbon production can result in a material with enhanced structural and chemical properties, improving enzymatic immobilization, catalytic efficiency, and operational stability, even under multiple cycles of use and harsh reaction conditions.^20^ The main objective is to evaluate the production of activated carbon from raw and fermented cocoa bean shells (CBS) and to investigate its application as a support for lipase immobilization in the enzymatic synthesis of hexyl butyrate, an ester with an apple-citrus aroma.^13,16^

To the best of the authors’ knowledge, this is the first study to develop and apply activated carbon derived from fermented cocoa bean shells (AC-FCBS) as a support for lipase immobilization aimed at catalyzing esterification reactions.^21,22^ This innovative approach provides a robust, sustainable, and scalable solution for large-scale applications, valorizes agro-industrial residues, and offers a cost-effective alternative to synthetic supports such as Diaion HP-20 (410 dollars per kilogram), contributing significantly to technological advancement in aroma production and aligning with circular economy principles. Moreover, this work is directly aligned with the United Nations Sustainable Development Goal 12 (Responsible Consumption and Production), promoting efficient use of resources, waste valorization, and circular economy principles.

## Materials and Methods

### Materials, Microorganism, and Inoculum

Amano Lipase PS from *Burkholderia cepacia* (APS) and the commercial support Diaion HP-20 (DIR) were used as comparative parameters and were purchased from Sigma-Aldrich Chemical Co. (St. Louis, MO, USA). The enzyme used in this study is Amano Lipase PS, a commercial enzyme obtained from Sigma-Aldrich. This lipase is derived from *Burkholderia cepacia* and has predefined specifications, including an activity of 30,000 U/g, an optimum pH of 7.0, and an optimum temperature of 50 °C, as indicated by the supplier. Extra virgin olive oil was acquired from a local market in Ilhéus, Bahia, Brazil. Butyric acid (99%) and hexanol (98%) were obtained from Synth (São Paulo, SP, Brazil). All other reagents were sourced from Vetec Química (São Paulo, SP, Brazil).

Cocoa bean shells (CBS), *Theobroma cacao*, were obtained from a cocoa processing unit in Itabuna, Bahia. To prepare the inoculum for SSF, the fungus *Aspergillus niger* ATCC 1004 was subcultured under aseptic conditions in a laminar flow hood. This filamentous fungus, obtained from the microorganism collection of the National Institute for Quality Control in Health (INCQS) at Fiocruz (strain no. 40018), was cultivated on Potato Dextrose Agar medium. After 7 days of incubation at 26 °C in a bacteriological incubator (SOLAB model SL 101), a spore suspension was prepared by scraping the medium’s surface with sterilized distilled water and glass beads under agitation. Spore counting was performed using a Neubauer chamber under a binocular microscope (BIOVAL L1000, São Paulo, Brazil).^23^

### Preparation of Fermented Biomass

The CBS were received in a dried state and subsequently processed using a Wiley knife mill (ACB LABOR, São Bernardo do Campo, SP, Brazil) to achieve a particle size of 2 mm. For the SSF process, 125 mL Erlenmeyer flasks were used, with CBS serving as the sole carbon source. Initially, 10 g of biomass were sterilized in an autoclave at 121 °C for 15 min and then inoculated with a spore suspension at a concentration of 10^–7^ spores per gram of substrate. The fermentations were carried out in BOD bacteriological incubators (SL 222, Solab, Piracicaba, Brazil) at 30 °C for 5 days. The moisture content was adjusted to 60% by adding autoclaved distilled water, and moisture levels were measured using an Aqualab analyzer (Shimadzu, Barueri, SP, Brazil).^24^

### Preparation of Activated Carbon

Two types of AC were produced: one from CBS and the other from the reutilization of fermented cocoa bean shells (FCBS). Both were produced following the methodology described in the literature.^14^ Both samples were treated with an activating agent, phosphoric acid, at a ratio of 2.5:1 g/g for 48 h at 105 °C in an oven (TE 393/1, Tecnal, São Paulo, Brazil). Subsequently, sim they were carbonized at 500 °C under a nitrogen flow of 50 mL/min for 1 h in a muffle furnace (3–550, Vulcan, São Paulo, Brazil). The resulting activated carbons, were then washed with deionized water at 60 °C until the pH reached 7.0, signaling the end of the washing process. Granulometric separation was performed using a 420 mesh sieve and stored in airtight containers. The yields of the activated carbons were calculated using eq 1.

1 R%=(cm/pm)×100

where *R* is the yield of activated carbon (%), cm is the mass of the obtained carbon (g), and pm is the mass of the precursor agri-food byproducts (g).

### Immobilization of APS on the Produced and Commercial Supports

#### Effect of APS Concentration on the Immobilization Support

Different concentrations (1, 3, 6, and 9 mg/g of support) were evaluated over 24 h to determine which concentration provided the best enzyme retention or a retention level similar to the synthetic support Diaion HP-20 (DIR). Immobilization via physical adsorption was performed following a methodology adapted from.^14^

The samples AC-CBS, AC-FCBS, and DIR (1 g) were immersed in 25 mL of 95% ethyl alcohol in beakers for 4 h at room temperature, then filtered under vacuum using a Prismatec system (Itu, SP, Brazil). The humidified supports (CBS and FCBS) were then added to 19 mL of an Amano Lipase PS enzyme solution (S-APS) prepared with 5 mM sodium phosphate buffer at pH 7 and homogenized at 200 rpm (Q816M20, Quimis, Diadema, SP, Brazil) at 25 °C for 12 h.^18^ After the immobilization process, the biocatalysts with APS were filtered under vacuum (Prismatec, Itu, SP, Brazil) and stored at 4 °C for 12 h before use.

#### Determination of Hydrolytic Activity

The titration methodology was performed using extra virgin olive oil with acidity up to 0.8%. To begin the process, 0.1 mL of the S-APS or the supernatant (resulting from the immobilization process) was added to an emulsion (19 mL) composed of 5% (w/v) oil and 3% (w/v) arabic gum in sodium phosphate buffer (100 mM, pH 7.0). This mixture was incubated at 37 °C and 200 rpm for 5 min, with the reaction time being determined after evaluating the initial rates. These standard conditions, set at 37 °C and pH 7.0, were used as a reference.^14^

The reaction was stopped by adding 10 mL of 95% ethanol, which not only halted the reaction but also facilitated the extraction of free fatty acids. The released fatty acids were then titrated with NaOH (0.3 M). Reaction blanks were prepared by adding ethanol before introducing the enzyme solution. For quantification, one unit of lipase was defined as the amount of enzyme capable of releasing 1 μmol of fatty acids per minute under the specified assay conditions. Enzymatic activity was expressed in units per gram of enzyme (U/g). The immobilization efficiency was calculated as the ratio of free enzyme activity to immobilized enzyme activity, as described in eq 2. All samples were performed in triplicate.

2 $$\mathrm{HA}\;\left(U/g\right)=\frac{\left(V_{a}-V_{b}\right)\times M_{\left(\mathrm{NaOH}\right)}\times 1000}{t\times m}$$

where HA = hydrolytic activity; *V*_a_ = volume of NaOH from the titrated sample; *V*_b_ = volume of NaOH from the titrated blank; *M*_(NaOH)_ = molarity of the standardized NaOH solution; *t* = reaction time; *m* (enzyme) = amount of S-APS used.

#### Sodium Dodecyl Sulfate Polyacrylamide Gel Electrophoresis (SDS-PAGE)

Electrophoresis was used to indirectly evaluate the efficiency of different concentrations in the enzymatic immobilization process by analyzing the supernatant after immobilization. A total of 20 μL of the supernatant was mixed with 5 μL of buffer solution containing 10% (w/v) glycerol, 5% (w/v) β-mercaptoethanol, 2.3% (w/v) SDS, and 0.0625 M Tris-HCl (pH 6.8). The sample was denatured in a thermocycler for 7 min and then loaded onto a 12.5% (w/v) SDS-polyacrylamide gel, following the method described by Laemmli (1970). Electrophoresis was performed at a constant voltage of 30 mA for 4 h using a GE Healthcare mini-gel system (Amersham Pharmacia Biotech, UK). After electrophoresis, the gels were stained with Coomassie Brilliant Blue and scanned using a Visioneer OneTouch scanner (OneTouch 8700, Visioneer, Burlington, USA).

### Characterization of Precursor Material, Supports, and Biocatalysts

#### Determination of Cellulose, Hemicellulose, and Lignin

The contents of cellulose, hemicellulose, and lignin in cocoa shells were determined according to the ref 25 methodology, adapted for lignocellulosic residues. Samples were oven-dried, ground to <1 mm, and analyzed for neutral detergent fiber (NDF), representing hemicellulose, cellulose, and lignin, and acid detergent fiber (ADF), which includes cellulose and lignin, using standard detergent solutions under reflux, followed by filtration and drying. Lignin was quantified via the Klason method, through hydrolysis of the ADF residue with 72% H_2_SO_4_, subsequent dilution to 3%, and reflux. Cellulose content was calculated as the difference between ADF and lignin, while hemicellulose was obtained by the difference between NDF and ADF. All analyses were performed in triplicate and expressed on a dry weight basis (% d.w.) to ensure accuracy and reproducibility.

#### Surface Area Analysis by N_2_ Adsorption

Surface area analysis was performed using a BELSORP II-MINI surface area analyzer (BEL, Japan) at 77 K at the Center for Strategic Technologies of the Northeast, Recife, Brazil. The surface area, porosity, and pore volume of the samples were determined using the BET^16,18,26^ and BJH^27^ methods.

#### Surface Morphology

Scanning electron microscopy (SEM) analysis was conducted using a Quanta 250 scanning electron microscope (Peabody, Massachusetts, USA) at the Scanning Microscopy Center, Santa Cruz State University, Ilhéus, Brazil. This analysis was performed to examine the surface morphology of the samples. The acceleration voltage was set at 15 kV. Prior to imaging, the samples were coated with gold and securely mounted on carbon conductive tape. No mechanical or chemical treatments were applied to ensure uniform characterization.

#### Fourier Transform Infrared Spectroscopy (FTIR)

Fourier Transform Infrared Spectroscopy (FTIR) was used to analyze the chemical structure, functional groups, and corresponding bond vibrations of the samples. The measurements were performed using a Cary 630 FTIR spectrometer (Agilent Technologies, Santa Clara, USA) at the Advanced Materials Research and Innovation Laboratory, Santa Cruz State University, Ilhéus, Brazil. The spectra were acquired using an attenuated total reflectance (ATR) cell equipped with a deuterated triglycine sulfate (DTGS) detector. Data were recorded in the spectral range of 4000 to 500 cm^–1^, with a resolution of 4 cm^–1^ and 64 scans.

#### Thermogravimetric Analysis (TG/DTG)

The thermal properties of the samples were analyzed using a Q500 thermogravimetric analyzer (TA Instruments, New Castle, USA) at the Advanced Materials Research and Innovation Laboratory, Santa Cruz State University, Ilhéus, Brazil. This instrument measures variations in sample mass in response to temperature and ambient gas conditions. Thermograms were recorded under a nitrogen atmosphere, with a heating rate of 10 °C/min over a temperature range of 25 to 800 °C. Additionally, derivative thermogravimetry (DTG) was used to identify the temperatures at which significant thermal events occurred, which were analyzed based on the mass loss observed in the TGA thermograms.

#### Application of Biocatalysts in Aroma Compound Production

The synthesis of an apple-citrus aroma ester was performed in sealed Duran flasks containing butyric acid and hexanol dissolved in heptane. The substrates were used at a 1:1 v/v molar ratio, with a total reaction volume of 25 mL. The reactions were carried out using the biocatalysts APS-AC-CBS, APS-AC-FCBS, and APS-DIR at a concentration of 10% w/v, under agitation at 200 rpm and a temperature of 40 °C for 30 h (Q816M20, Quimis, Diadema, Brazil).

The reaction progress was monitored by collecting 200 μL samples at specific time intervals to quantify the residual butyric acid concentration via titrimetry. The conversion rate was calculated using eq 3.^28^

3 $$\mathrm{Conversion}\;\%=\frac{A_{\left(\mathrm{initial}\right)}-A_{\left(\mathrm{final}\right)}}{{\mathrm{AH}}_{\left(\mathrm{final}\right)}}\times 100$$

where *A*_initial_ and *A*_final_ are the initial and final fatty acid concentrations in the reaction medium (mM).

#### Stability Tests of Biocatalysts

For the stability and reuse tests, the same conditions described in Section 2.6 were applied. A total of ten cycles were performed, and at the end of each cycle, the biocatalysts were removed from the reaction medium and washed with cold hexane.^29^

#### Gas Chromatography GC-FID

The hexyl butyrate ester synthesis was analyzed by gas chromatography, compared to a standard, using a SHIMADZU GC-2010 Plus (Kyoto, Japan) equipped with a Flame Ionization Detector (FID) and a Restek capillary column (0.25 mm internal diameter, 0.25 μm film thickness). The column and FID temperatures were set at 260 and 250 °C, respectively. The temperature program was initiated at 100 °C, held for 1 min, then increased to 170 °C at a rate of 5 °C/min and held for an additional 2 min. The total run time was 17 min, and the retention time for hexyl butyrate was 5.7 min.

#### Statistical Analysis

All analyses were conducted in triplicate, and the results are expressed as the mean ± standard deviation. Statistical comparisons were performed using Tukey’s test (*p* < 0.05) with the Statistical Analysis System (SAS) version 8.0 and Origin Pro 8.0.

## Results and Discussion

### Yield of Activated Carbons

Bromatological analyses revealed that cocoa bean shells (CBS) possess a significant lignocellulosic composition, comprising lignin, cellulose, and hemicellulose. To assess the impact of solid-state fermentation (SSF), the composition was measured before and after the process: prior to fermentation, lignin (23.23%), cellulose (21.38%), hemicellulose (5.20%); following fermentation, lignin (24.60%), cellulose (22.78%), hemicellulose (6.28%). The slight increase observed in these components can be attributed to partial disruption of the lignocellulosic structure during SSF, which exposes or concentrates structural fractions of the biomass. This composition strongly influences carbonization behavior and the properties of the resulting activated carbon. The high lignin content a thermally stable and structurally complex polymer facilitates micropore formation during pyrolysis, whereas cellulose and hemicellulose contribute to the carbon framework, mainly through volatile release. Therefore, the unique chemical composition of CBS, together with the effects of SSF, plays a key role in determining both the yield and the porous structure of the activated carbon produced.^20^

Previous studies on lignocellulosic agri-food byproducts with a similar lignin content (24.88%) reported surface areas of approximately 760 m^2^/g.^30^ Previous studies have shown that changes in the lignin content and structure of lignocellulosic residues can significantly influence the yield and characteristics of the activated carbon produced.^30^ Although high lignin content contributes to the formation of micropores, it can hinder the development of large surface areas due to its recalcitrant nature. In this context, bioprocessing with filamentous fungi has emerged as a promising strategy, as it can induce modifications in the lignocellulosic matrix, such as partial degradation or selective removal of lignin. These structural changes can enhance carbonization and improve the final properties of the activated carbon, including porosity and surface structure.

Moreover, controlled lignin reduction promotes structural rearrangements that enhance the development of micro- and mesopores, thereby increasing the surface area. Thus, the use of bioprocesses adds value to lignocellulosic agri-food byproducts and offers a sustainable approach for producing activated carbon with enhanced properties, making it suitable for industrial applications.^16^

Based on the data from Table 1, it is noted that AC-CBS and AC-FCBS carbon presented yields of 32% and 42%, respectively. This yield, when above 30%, suggests remarkable efficiency in the production process, which is crucial for companies aiming to maximize production and reduce costs. The comparison with the yield of solid wood carbon contextualizes the process’s effectiveness, indicating a potential competitive advantage for the industry. Such information is valuable for companies seeking process optimization and ensuring a competitive market position.^31^

**Table 1:** Gravimetric Yield of Activated Carbons Obtained from 100 g of Raw Cocoa Bean Shells (AC-CBS) and Fermented Cocoa Bean Shells (AC-FCBS), Expressing the Percentage of Material Obtained after Carbonization^a^

Support	Yield (%)
AC-CBS	32.00 ± 0.02^a^
AC-FCBS	42.00 ± 0.01^b^

a Means followed by the same letter do not differ statistically from each other according to the Tukey test (*p* < 0.05). Mean values ± standard deviation.

Temperature, in addition to the precursor material’s composition, is a key factor in the yield of activated carbons. Generally, higher carbonization temperatures (600–700 °C) lead to reduced AC yield but simultaneously increase the release rate of liquids and gases.^32^ Therefore, although higher temperatures result in better quality carbon, they are also associated with a decrease in yield.^33^

This study explores the feasibility of reusing agri-food byproducts from a bioprocess induced by filamentous fungi, using the SSF technique, to produce activated carbons with potential for industrial applications. The structural modifications in the fibers of FCBS, such as the degradation of amorphous components and lignin, can contribute to reducing lignin recalcitrance. This reduction facilitates carbon production at lower temperatures, thereby decreasing operational costs and yielding a material with promising characteristics, including high adsorptive capacity.^34^

These enhanced textural properties, such as increased surface area and porosity, are of particular interest for the application of this activated carbon (AC) as a support for enzyme immobilization in processes aimed at the biocatalysis of high-value industrial compounds. Although still under investigation, these advances suggest that the produced material can meet both performance and economic viability requirements for catalytic applications.^20^

### Effect of APS Lipase Concentration on the Support

Optimizing the maximum enzyme load adsorbed by the support is essential for improving immobilization efficiency and biocatalyst performance. Therefore, the lipase concentration was varied to determine the optimal amount for the proposed system, as shown in Table 2.

**Table 2:** Maximum Relative Yield of Enzymatic Activity of Amano PS Lipase (APS) Immobilized on Different Supports (APS-AC-CBS, APS-AC-FCBS, and APS-DIR)^a^

	Relative activity yield (%)			
Biocatalysts	1 mg/g	3 mg/g	6 mg/g	9 mg/g
APS-AC-CBS	79 ± 0.30^a^	72 ± 0.11^a^	45 ± 0.19^a^	59 ± 0.31^a^
APS-AC-FCBS	81 ± 0.12^a^	82 ± 0.16^a^	53 ± 0.20^a^	61 ± 0.22^ab^
APS-DIR	99 ± 0.11^b^	85 ± 0.20^b^	80 ± 0.21^b^	77 ± 0.041^b^

a Means followed by the same letter in each column do not differ statistically from each other according to the Tukey test (*p* < 0.05). Mean values ± standard deviation.

At an APS loading of 1 mg per gram of support, the maximum relative hydrolytic activities observed were 79%, 81%, and 99% for APS-AC-CBS, APS-AC-FCBS, and APS-DIR, respectively. When the APS loading increased to 3 mg per gram of support, the relative activities were 72%, 82%, and 85%, respectively. Data analysis indicates that synthetic supports exhibit superior efficiency at an APS loading of 1 mg/g (Figure 1a). However, at 3 mg/g, natural supports, particularly APS-AC-FCBS, demonstrate efficiency comparable to that of the synthetic support (APS-APS-DIR) (Figure 1b).

**Figure 1:**
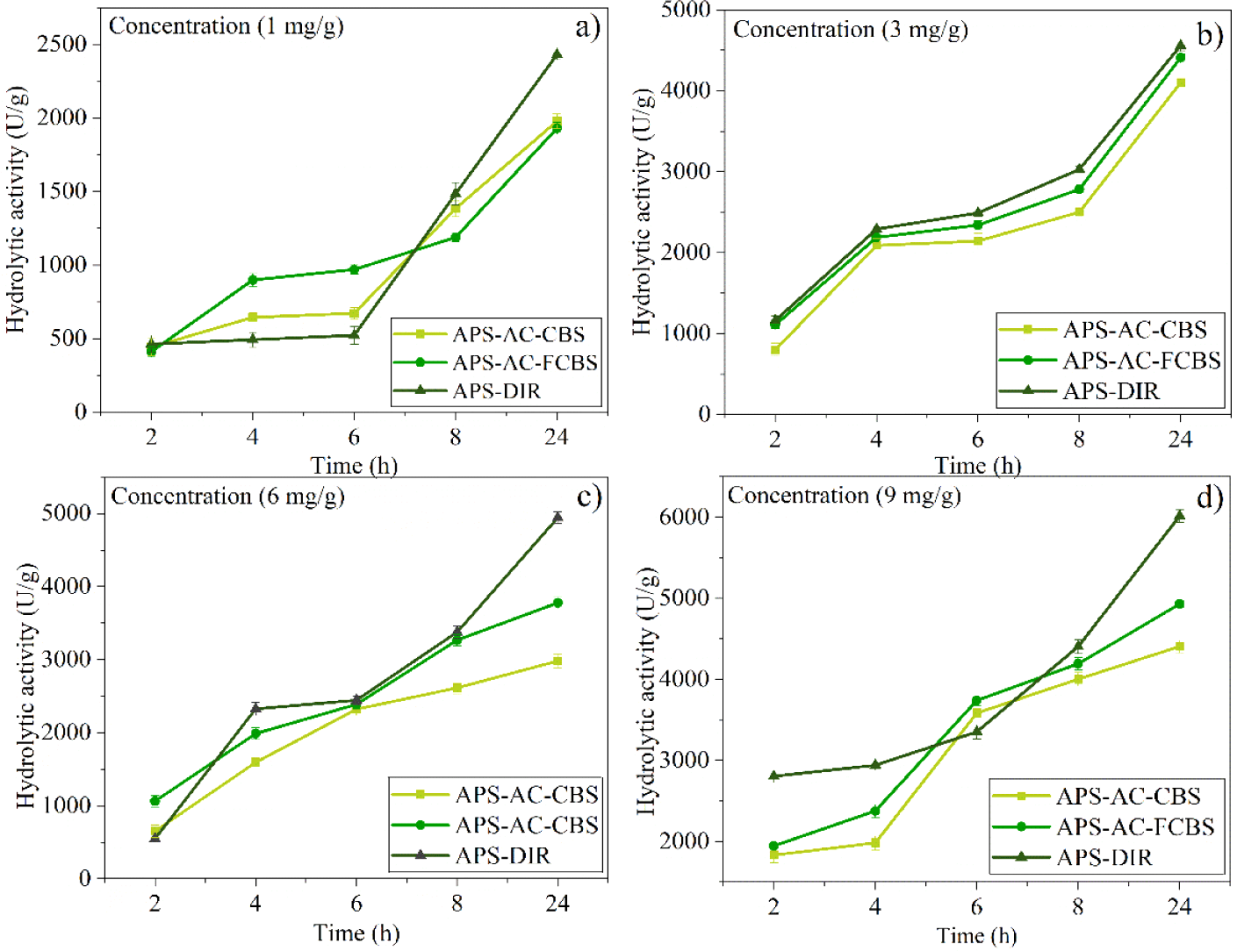
Hydrolytic activity of Amano PS lipase (*Burkholderia cepacia*) immobilized on activated carbons (APS-AC-CBS and APS-AC-FCBS) and Diaion HP-20 resin (APS-DIR) at different enzyme/support concentrations: (a) 1 mg/g, (b) 3 mg/g, (c) 6 mg/g, and (d) 9 mg/g.

Controlling enzyme concentration is a critical factor in the immobilization process, ensuring the near-complete fixation of a low enzyme load. This outcome can be attributed to the synergy of multiple factors, including the adequate availability of binding sites on the support, favorable interactions between the enzyme and the immobilization material, and the application of technically efficient immobilization methods. Proper enzyme concentration control not only optimizes enzyme utilization but also enhances stability and improves the biocatalyst’s reusability, which directly impacts industrial process efficiency.^16^

Moreover, immobilization yield and expressed activity were analyzed to provide a more accurate definition of the immobilization process, as recommended in recent studies.^18,35^ These parameters confirmed that APS-AC-FCBS exhibited improved efficiency due to its higher porosity and surface area, acquired through biomass fermentation prior to AC production.

Efficient enzyme immobilization is particularly crucial in industrial applications such as biofuel production, the synthesis of high-value-added chemical compounds, wastewater treatment, and the food sector. These applications benefit from more sustainable and cost-effective processes. Careful enzyme concentration adjustments ensure maximum catalytic performance, minimize waste, and enhance the economic viability of biocatalysis-based technologies.^36^

At concentrations of 6 mg/g and 9 mg/g, as detailed in Table 2 and illustrated in Figure 1c and d, enzyme adsorption decreased by approximately 27%, 29%, and 5% on APS-AC-CBS, APS-AC-FCBS, and APS-DIR supports, respectively, at 6 mg/g. A similar trend was observed at 9 mg/g.

This behavior is consistent with findings from a 2008 study on the immobilization of *Candida rugosa* lipase on green coconut fiber via physical adsorption. In that study, the decline in activity at higher enzyme loadings was attributed to the formation of enzyme multilayers, which blocked or inhibited access to active sites as enzyme concentration increased.^37^

At higher enzyme loadings (6–9 mg/g), a significant reduction in hydrolytic activity was observed, with decreases of approximately 27% for APS-AC-CBS, 29% for APS-AC-FCBS, and 5% for APS-DIR at 6 mg/g, and a similar trend at 9 mg/g. This behavior is consistent with findings from a 2008 study on the immobilization of *Candida rugosa* lipase on green coconut fiber at high enzyme concentrations, attributed to multilayer formation on the support surface. Such multilayers may block or restrict access to the enzyme’s active sites, preventing efficient substrate interaction.^37^

In addition to multilayer formation, other mechanisms can explain this decline. Literature reports indicate that steric hindrance and diffusional limitations become increasingly relevant at high enzyme loadings, as densely packed enzyme molecules create barriers to substrate and product.^38^ Similarly, the immobilization of α-amylase on zinc sulfide-chitosan hybrid nanoparticles reduced catalytic efficiency, primarily due to steric effects and diffusional limitations that restrict substrate access to the enzyme’s active site.^39^ Moreover, adsorption site saturation at higher enzyme concentrations can compromise the effective loading capacity of the support, further lowering activity recovery.^40^

The novelty of this study lies in addressing these well-known limitations by introducing a bioprocessing step prior to activated carbon production. Solid-state fermentation of cocoa bean shells significantly enhanced the textural properties of the resulting material, yielding AC-FCBS with higher surface area and porosity than nonfermented precursors. These improvements alleviate diffusional barriers and reduce active-site blocking, thereby sustaining higher catalytic performance even at moderately elevated enzyme concentrations.^41^

Notably, APS immobilized on AC-FCBS achieved competitive conversion yields at only 3 mg/g of enzyme, comparable to the commercial resin Diaion HP-20, while using substantially lower enzyme amounts than those reported in previous studies (40 mg of *Candida rugosa* lipase required for ∼89% conversion).^37^

Enzyme immobilization using agro-food waste-derived carbons support these findings, emphasizing that biomass pretreatments such as fermentation enhance structural properties, improve adsorption efficiency, and mitigate diffusional and steric constraints at higher enzyme loadings. Therefore, the introduction of fermentation as a valorization step not only distinguishes AC-FCBS from other agro-waste-based activated carbons but also provides a sustainable and economically viable support with superior catalytic performance.^16^

However, unlike the study by Brígida,^37^ the present work highlights the importance of biomass bioprocessing before the AC production aiming the enhancement of support performance. This processing significantly increases the material’s surface area and porosity, enhancing enzyme adsorption capacity, reducing active site blockage, and improving catalytic efficiency even at high enzyme concentrations.

This study aligns with research on enzyme immobilization on AC derived from Spondias mombin (cajá) seeds, where increased enzyme loading was associated with diffusional limitations. These limitations create a diffusion barrier for substrates and products and introduce steric hindrance, which explains the reduction in enzymatic activity at high concentrations.^42^ Additionally, previous studies suggest that this phenomenon may result from medium saturation, which compromises adsorption capacity and, consequently, the efficiency of the support material.^43^

In the context of this study, the support material exhibits distinct characteristics due to the applied bioprocessing. This bioprocessing enhances the material’s surface area and porosity, resulting in a superior raw material for the development of activated carbon that mitigates the effects of saturation and diffusional barriers. These improvements increase enzyme adsorption efficiency, even at high concentrations, thereby minimizing negative impacts and enhancing overall catalytic performance.

The maximum enzyme loading capacity of the supports can be attributed to both the inherent and acquired properties of the materials, given that activated carbons naturally possess a porous structure and extensive surface area.^44^ Accordingly, the amano lipase PS immobilized on activated carbon derived from fermented cocoa bean shells (APS-AC-FCBS), with an enzyme concentration of 3 mg/g, exhibits a relative activity comparable to that of the synthetic support, amano lipase PS immobilized on Diaion HP-20 resin (APS-DIR). In addition to its environmentally friendly nature, APS-AC-FCBS offers significant advantages over APS-DIR. Its production utilizes an abundant, low-cost agri-food byproduct, unlike Diaion HP-20, which has an average price of 410 dollars per kilogram. Thus, APS-AC-FCBS helps reduce raw material costs and promotes the circular economy. Furthermore, biomass bioprocessing enhances the support’s surface area and porosity, improving adsorption efficiency and minimizing diffusional limitations—critical factors in industrial applications. Consequently, naturally derived AC presents a more sustainable and economical alternative for industries aiming to optimize catalytic processes without compromising performance.

As shown in Figure 1, an enzyme loading of 3 mg/g was selected as the optimal condition for subsequent applications, as it provides the best balance between recovered activity and immobilization yield. This enzyme concentration closely aligns with those used in real industrial processes for aroma production via enzymatic pathways, as reported in patents from companies in this sector. Thus, APS-AC-FCBS stands out not only for its sustainability but also as a cost-effective, high-performance alternative capable of meeting industrial demands for efficient and competitive solutions in the aroma market.

### Sodium Dodecyl Sulfate Polyacrylamide Gel Electrophoresis (SDS-PAGE)

The APS enzyme was identified as a band with an approximate molecular weight of 33 kDa, as shown in Figure 2. The molecular weight of the APS enzyme typically ranges between 33 and 37 kDa.^45^ When analyzing different concentrations, a decrease in band intensity was observed compared to the well containing the preimmobilization S-APS. This suggests that a significant portion of the enzyme was successfully immobilized onto the supports, corroborating previous findings and demonstrating the efficiency of AC as an enzyme immobilization support.

**Figure 2:**
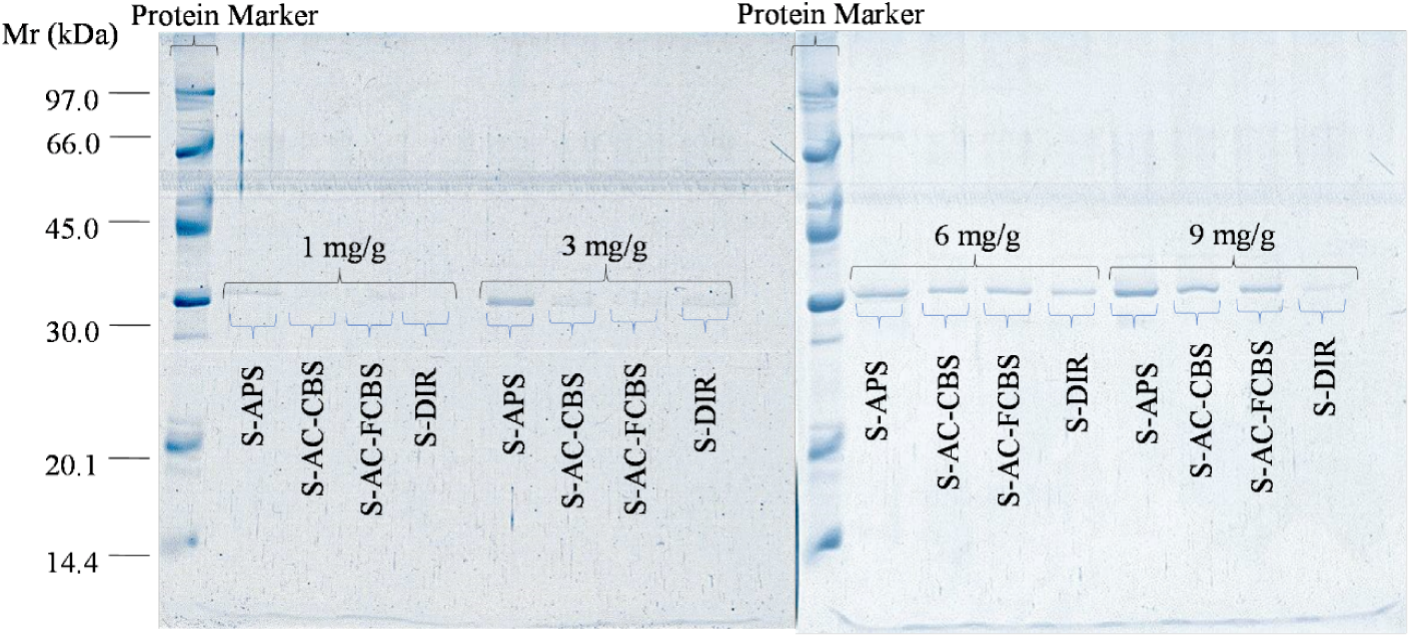
SDS-PAGE electrophoresis profile of Amano Lipase PS solution (S-APS), comparing the solutions after immobilization on different supports: activated carbon from cocoa bean shells (S-AC-CBS), activated carbon from fermented cocoa bean shells (S-AC-FCBS), and Diaion HP-20 resin (S-DIR), at concentrations of 1, 3, 6, and 9 mg/g.

Notably, the AC-FCBS, particularly at a concentration of 3 mg/g, exhibited remarkable enzyme adsorption capacity. This was evidenced by the pronounced reduction in the color intensity of the supernatant (S-AC-FCBS) following immobilization, further confirming the material’s effectiveness in enzyme retention.

### Characterization of the Precursor Material, Supports, and Biocatalysts

#### Surface Area Analysis by N_2_ Adsorption

The characterization of adsorbent capacity before and after the immobilization of APS is crucial for assessing the performance of the developed activated carbons as catalytic supports. Table 3 provides data on the specific surface area, total pore volume, and average pore diameter of these materials. These structural properties are key in determining the interaction between the adsorbent materials and target compounds, helping to clarify the underlying adsorption mechanisms in comparison to other systems and pollutants. According to the classification established by the International Union of Pure and Applied Chemistry (IUPAC), porous materials are categorized based on pore size: microporous (<2 nm), mesoporous (2–50 nm), and macroporous (>50 nm).^46,47^

**Table 3:** Textural Properties of Activated Carbons (AC-CBS and AC-FCBS) and Their Corresponding Biocatalysts (APS-AC-CBS and APS-AC-FCBS), Determined from N_2_ Adsorption–Desorption Isotherms at 77 K^a^

Sample^b^	Sg (m^2^/g)	Dp (nm)^c^	*V*_meso_ (cm^3^/g)^d^	*V*_micro_ (cm^3^/g)^e^
AC-CBS	1050.30	7.282	1.105	0.144
AC-FCBS	1158.08	6.505	1.122	0.145
APS-AC-CBS	445.07	8.042	0.581	0.050
APS-AC-FCBS	249.38	8.607	0.374	0.029

a Parameters include BET surface area (m^2^/g), pore volume (cm^3^/g), and average pore diameter (nm).

b BET surface area.

c Dp: Average pore diameter.

d *V*_meso_: Mesopore volume.

e *V*_micro_: Micropore volume.

Figure 3 shows the nitrogen (N_2_) adsorption and desorption isotherms of the activated carbons (AC-CBS, AC-FCBS) and biocatalysts (APS-AC--CBS, APS-AC-FCBS). According to IUPAC (1982), these isotherms can be classified as type IV, indicating the occurrence of capillary condensation, which is characteristic of mesoporous materials. This type of isotherm demonstrates the formation of a monolayer on the surface, followed by multilayer adsorption until reaching the inflection point and eventual isotherm saturation. The initial slope observed at low *P*/*P*_o_ values corresponds to monolayer coverage, while the second slope represents the transition to multilayer adsorption and capillary condensation.

**Figure 3:**
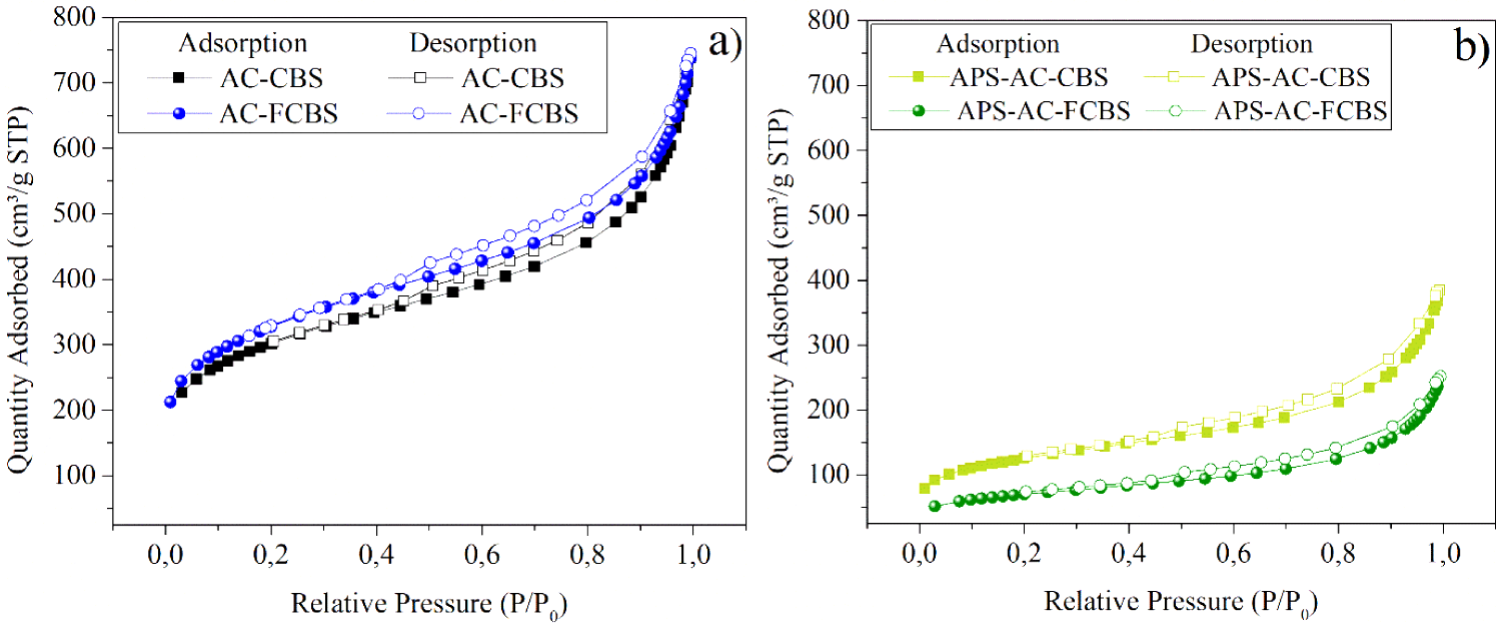
Nitrogen adsorption/desorption isotherms of: (a) activated carbons derived from cocoa bean shell (AC-CBS) and fermented cocoa bean shell (AC-FCBS), and (b) the corresponding biocatalysts immobilized with *Aspergillus* lipase (APS-AC-CBS and APS-AC-FCBS). The isotherms indicate the textural properties and adsorption behavior of the materials.

The presence of a more pronounced hysteresis loop at high relative pressures suggests a mixed pore structure in the material, consisting of both micropores and mesopores, as observed in the evaluated activated carbons (Brito et al., 2017;^42^ Kumar and Jena, 2016 ^48^). The hysteresis behavior shown in Figure 3 can be classified as type H3. According to IUPAC (1982), this type of hysteresis is typically observed in aggregates of plate-shaped particles, leading to the formation of slit-shaped pores.

In immobilization processes via adsorption, it is desirable for the adsorbent to exhibit effective interaction between mesopores and micropores. Mesopores facilitate the rapid diffusion of molecules within the carbon structure, thereby enhancing access to micropores, where adsorption predominantly occurs.^49^ It is also noted that the volume of N_2_ adsorbed by the AC-FCBS sample is higher compared to the AC-CBS sample, indicating greater pore volume availability for the adsorption process and, consequently, a larger surface area. These characteristics suggest that AC-FCBS has a higher adsorption capacity during the immobilization process.

Fermentation of the precursor biomass is expected to mitigate these limitations by modifying both the textural and chemical properties of the activated carbon. During solid-state fermentation, selective degradation of lignin and partial depolymerization of hemicellulose increase porosity and expose functional oxygenated groups on the carbon surface. These changes improve accessibility to mesopores, favor enzyme immobilization, and reduce nonspecific adsorption of substrates or products. Consequently, the fermented-derived activated carbon (AC-FCBS) provides higher selectivity, minimizes pore blockage, and maintains catalytic activity over repeated cycles. Compared to the unfermented material, the bioprocessed support exhibits enhanced stability and efficiency, representing a more robust solution for large-scale applications.^50^

The textural parameters of the adsorbents are summarized in Table 3, highlighting that the activated carbons (AC-CBS, AC-FCBS) exhibited considerable surface areas of 1050.30 m^2^/g and 1158.08 m^2^/g, respectively. This result is attributed to the composition of the carbon precursor and the activation method employed. The results of the present study surpass those of previous studies in terms of surface area,^51,52^ which reported surface areas of 969 m^2^/g using cocoa bean shell as a precursor and 962 m^2^/g when phosphoric acid was used as an activating agent. The superior performance observed in this study may be attributed to optimizations in the synthesis procedures or modifications in experimental parameters, such as reaction time, temperature, or reagent concentration, which were adjusted to enhance material production efficiency.

Scientific literature indicates that lignocellulosic materials with higher cellulose content tend to produce activated carbons with larger surface areas, due to their lower resistance to thermal degradation compared to materials dominated by lignin.^53^ In the case of AC-FCBS, an 11% increase in surface area and a 2% increase in mesopore volume were observed. These results support the effectiveness of bioprocessing with filamentous fungi in carbon production, particularly when the precursor contains substantial amounts of lignin. During the bioprocess using the SSF technique, filamentous fungi such as *Aspergillus niger* promote partial lignin degradation, making it more accessible to the activating agent during the activation stage. As a result, the subsequent carbonization process can be conducted at moderate temperatures, as the lignin is more susceptible to chemical modification facilitated by the SSF process.^12^

The analysis of APS-AC-CBS and APS-AC-FCBS in Table 3 suggests a significant reduction in surface area and micro- and mesopore volumes, indicating greater enzyme immobilization on the support. This reduction is due to the occupation of space by the enzymatic material, leading to a decrease in the availability of surface area and porosity. Additionally, enzyme immobilization in the pores may result in partial or complete pore filling, contributing to the reduction in micro- and mesopore volumes. APS-AC-FCBS showed reductions of 56%, 64%, and 58% in surface area, mesopore volume, and micropore volume, respectively, compared to APS-AC-CBS, implying a lower amount of N_2_ adsorbed due to the higher enzyme content in the pores.^20^

The porous structure of AC plays a crucial role in the adsorption of molecules, as it restricts their dimensions and directly influences their interaction with the adsorbent material. The increase in surface area, as well as the enlargement of mesopore diameter and volume identified in APS-AC-FCBS compared to APS-AC-CBS, results in greater availability of the adsorbed material to interact with the pores. This improvement can be attributed to the fermentation process undergone by the precursor material.^54^ This observation is further supported by the more efficient interaction between APS-AC-FCBS and the enzyme during the immobilization process.

#### Scanning Electron Microscopy (SEM)

The micrographs reveal that the surface of the CBS used is compact, with rigid and ordered fibrils, and no noticeable changes in the morphology of the plant structure (phloem and xylem), as indicated by the arrows in Figure 4a. These images were obtained using scanning electron microscopy (SEM), as shown in Figure 4.

**Figure 4:**
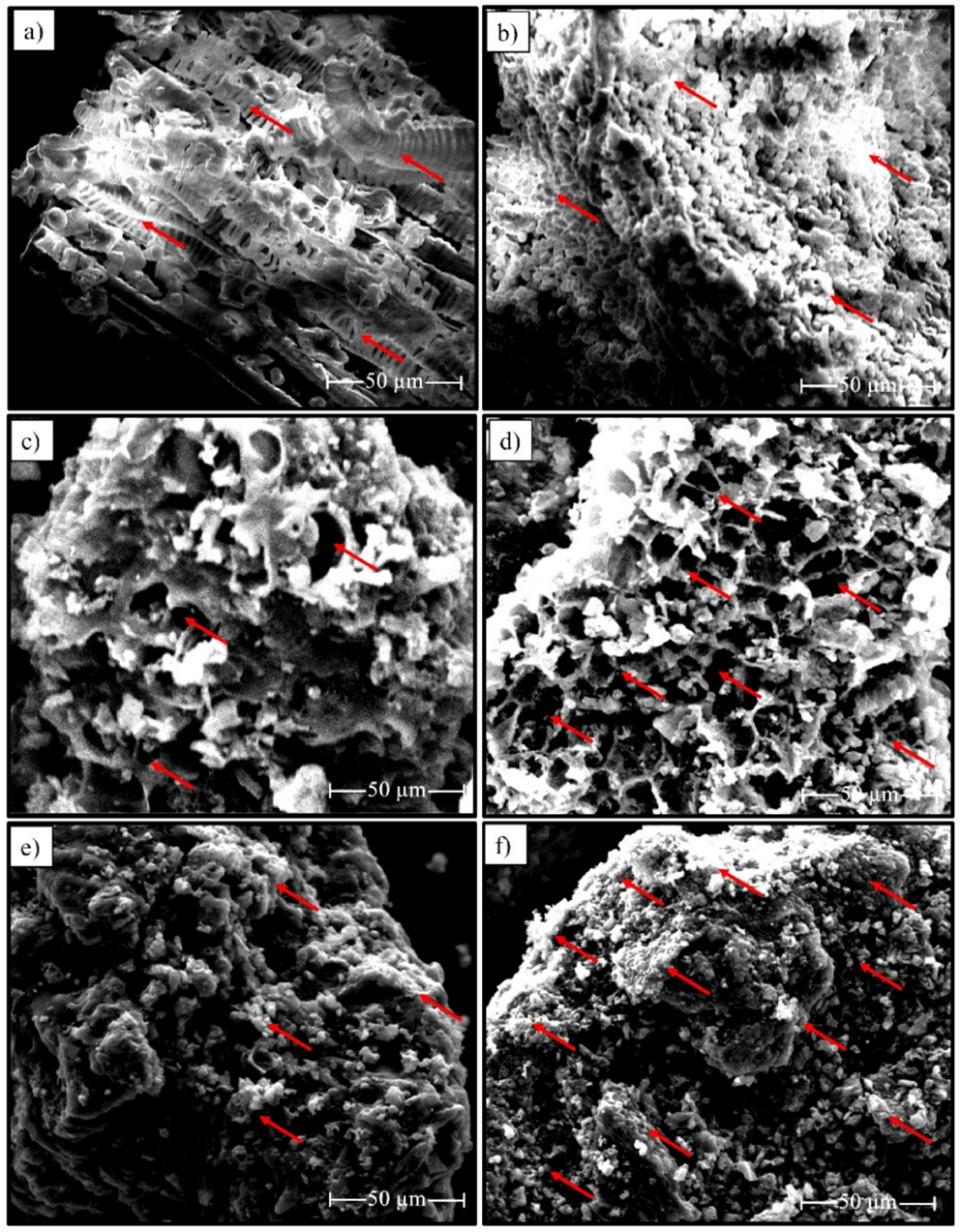
Scanning Electron Microscopy (SEM) micrographs showing the morphological features of: (a) raw cocoa bean shell (CBS), (b) fermented cocoa bean shell (FCBS), (c) activated carbon from cocoa bean shell (AC-CBS), (d) activated carbon from fermented cocoa bean shell (AC-FCBS), (e) biocatalyst obtained from immobilization of lipase on AC-CBS (APS-AC-CBS), and (f) biocatalyst obtained from immobilization of lipase on AC-FCBS (APS-AC-FCBS).

However, during the SSF process, the surface of the CBS becomes colonized by filamentous fungi *Aspergillus* niger ATCC 1001, as indicated by the arrows in Figure 4b. This SSF process caused significant structural changes due to the partial degradation of the surface lignin.^55^

The micrographs of the activated carbons (APS-AC-CBS and APS-AC-FCBS), shown in Figure 4c and d, support this analysis. Using FCBS as raw material, the carbonaceous material (Figure 4d) loses the compact structure observed in Figure 4c shows greater porosity, as highlighted by the arrows. The degradation of lignin by filamentous fungi during SSF plays a critical role in the structural modification of the lignocellulosic material, facilitating the depolymerization and partial removal of lignin. This results in greater exposure of cellulose and hemicellulose, contributing to the formation of a more porous and reactive structure.

Such structural changes make the fermented material highly suitable for AC production, potentially improving adsorption performance due to the increased surface area and chemical functionalization. This transformation enhances the efficacy of activating agents and allows the use of lower temperatures to obtain activated carbons with high surface area and porosity.^18^ The removal of lignin also improves the access of activating agents to cellulose and hemicellulose, which are responsible for imparting textural properties to activated carbons and enhancing their efficiency.^14,56^

Studies on activated carbons produced through various carbonization processes have shown structural modifications with the formation of micropores. Initially, these changes were attributed to cellulose degradation, followed by partial lignin degradation due to its higher stability. However, comparing these findings with the results from this study, its possible to conclude that SSF fermentation significantly influences lignin degradation, leading to improvements in the textural properties.^57^ Other studies exploring the modification of lignocellulosic materials also support this trend, noting that these materials lose their compact structure and exhibit a marked increase in surface area.^58,59^

After analyzing the micrographs of the produced biocatalysts (Figure 4e and f), the presence of the immobilized enzyme on the activated carbons is evident. In Figure 4e (APS-AC-CBS), enzyme immobilization appears to occur predominantly inside the pores. In contrast, Figure 4f (APS-AC-FCBS) shows enzyme immobilization both within the pores and on the external surface, covering nearly all surface pores. This configuration likely contributed to the higher relative activity yield observed for APS-AC-FCBS, as shown in Table 1.

#### Fourier Transform Infrared Spectroscopy (FTIR)

FTIR spectral analysis enabled the observation of chemical changes resulting from SSF, providing valuable insights into the suitability of AC-CBS and AC-FCBS as supports for enzyme immobilization, particularly regarding their chemical composition. The FTIR spectra of agri-food byproducts, activated carbons, and biocatalysts revealed variations in transmittance after SSF, indicating alterations in the chemical constituents of CBS, AC-CBS, and APS-AC-FCBS, as well as the presence of fungal biomass (Figure 5).

**Figure 5:**
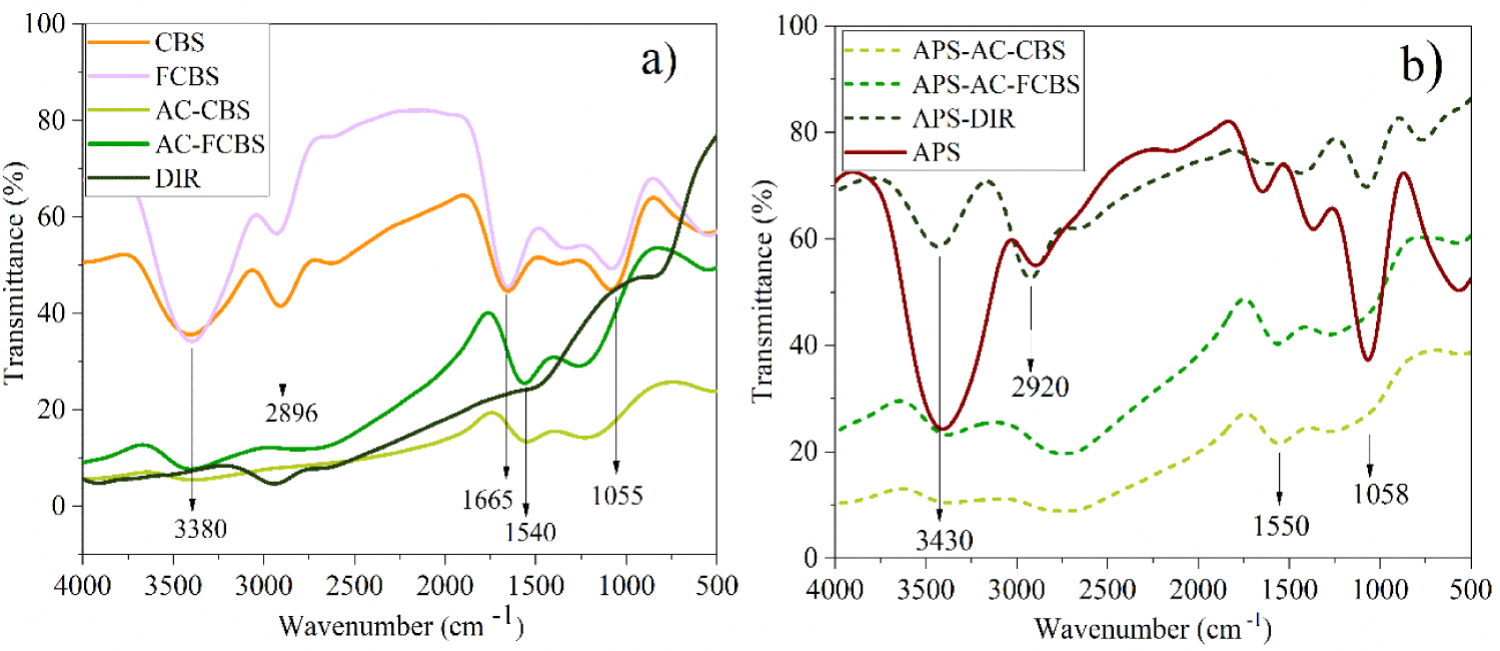
FTIR spectra of the analyzed materials: (a) cocoa bean shells (CBS), fermented cocoa bean shells (FCBS), activated carbon from cocoa bean shells (AC-CBS), activated carbon from fermented cocoa bean shells (AC-FCBS), Diaion HP-20 resin (RDI); (b) biocatalysts (APS-AC-CBS, APS-AC-FCBS, and APS-DIR), and Amano lipase PS (APS).

These findings were corroborated by BET and SEM analyses, which also revealed structural modifications in the agri-food byproducts after SSF, thereby enhancing the textural characteristics of the produced supports. In Figure 5a, it is evident that the biomass (CBS and FCBS) contains a variety of functional groups, including esters, ethers, alcohols, aldehydes, ketones, phenols, and carboxylic acids. Notably, the presence of an intense band at 3380 cm^–1^ can be attributed to the stretching vibrations characteristic of hydroxyl (−OH) groups in cellulose. Additionally, the band at 2896 cm^–1^ corresponds to the axial deformation of C–H bonds, characteristic of cellulose, hemicellulose, and lignin. The distinct bands at 2920 cm^–1^ and 2888 cm^–1^ are associated with the symmetric vibrational stretching of C–H bonds.^20^ The band at 1665 cm^–1^ is linked to the vibration of the C=O group present in carboxymethylcellulose, while a prominent peak at 1028 cm^–1^ corresponds to the stretching of the C–O bond in cellulose, hemicellulose, and lignin—key features of lignocellulosic materials. The changes in intensity, wavelength shifts, or disappearance of these bands after carbonization (CBS and FCBS) indicate structural modifications in the precursor material.^16^

In the spectra of AC (CBS and FCBS) shown in Figure 5a, vibrations related to the carbon skeleton are observed at 1540 cm^–1^, corresponding to the symmetric stretching of the C=C bond in aroma rings a distinctive feature of carbonaceous materials.^60^ Additionally, bands around 1230 cm^–1^ can be attributed to the stretching of hydrogen bonded to P=O in phosphate esters, the O–H bond in POC, or P=OOH bonds incorporated on the material’s surface during activation with H_3_PO_4_.^13^ These characteristic bands are not present in the DIR as it consists of a resin that lacks the functional groups typical of carbonaceous materials. In the spectrum of the free enzyme, distinctive protein adsorption bands are detected, particularly the amino (R-NH) and carboxyl (R-CO−) groups, which appear in the spectral regions of 1550 cm^–1^ and 1300 cm^–1^, respectively.

A comparison between the spectra in Figure 5a (before immobilization) and Figure 5b (after immobilization) reveals changes in the chemical composition of the carbon material’s surface, highlighting the complex interaction between the APS lipase and the support. These modifications provide clear evidence of enzyme adhesion to the support, as indicated by the detection of a band at 3430 cm^–1^, which corresponds to the overlap of the stretching vibrations of the carbon’s OH groups with the NH stretching vibrations of the enzyme. Additionally, a prominent band at 1058 cm^–1^ is observed, associated with the characteristic vibrations of the amino groups present in the lipase structure.^61^

#### Thermogravimetric Analysis (TG/DTG)

The TG and DTG curves illustrate the thermal stability of the materials (Figure 6). Weight loss was evaluated across three regions. In region I (0 to 150 °C) (Figure 6a and c), mass loss corresponds to the elimination of volatiles and water, with approximately 10% loss for CBS and FCBS, 15% for DIR, 22% for AC-CBS, and 25% for AC-FCBS, as shown in Figure 6a. On the other hand, the biocatalysts exhibited a mass loss of approximately 3% for APS-DIR, 7% for APS, and 18% for the natural biocatalysts APS-AC-CBS and APS-AC-FCBS (Figure 6c). The precursor materials showed lower mass loss compared to the activated carbons, indicating greater hydrophobicity. As porous materials, activated carbons tend to adsorb more moisture from the environment, leading to a higher evaporation-induced mass loss. A similar trend is observed when comparing synthetic and natural biocatalysts. The lower mass loss observed after immobilization can be attributed to the stronger retention of water molecules, due to the presence of hydrophilic functional groups (NH^2–^) on the APS surface. These groups promote stronger interactions with water, hindering its evaporation during thermal analysis in the 0 to 150 °C range. Additionally, it is possible that organic groups present in the APS structure interact with the functional groups of activated carbon, contributing to a more stable matrix and reduced susceptibility to moisture release.

**Figure 6:**
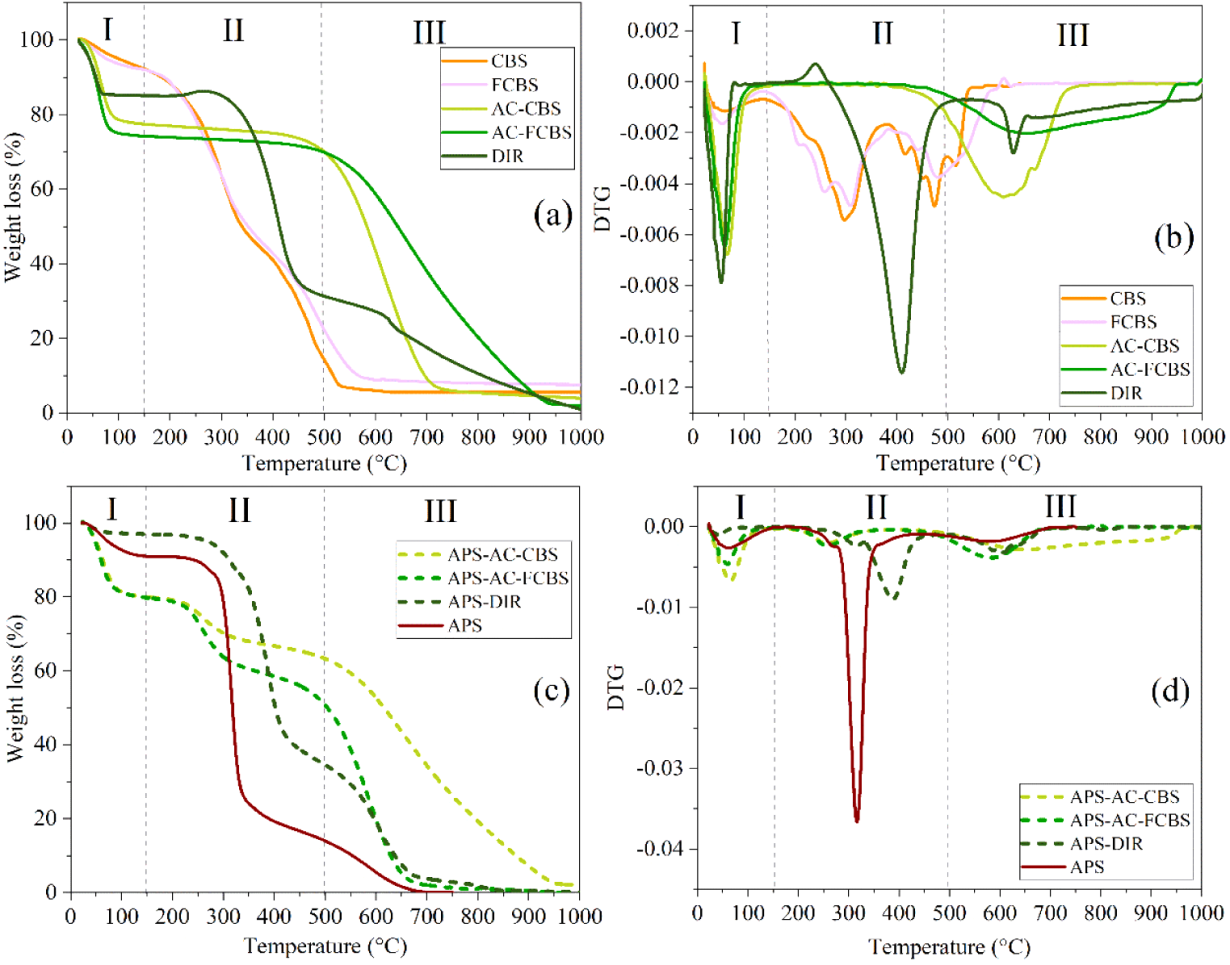
Thermal analysis of the analyzed materials: (a and b) cocoa bean shells (CBS), fermented cocoa bean shells (FCBS), activated carbon from cocoa bean shells (AC-CBS), activated carbon from fermented cocoa bean shells (AC-FCBS), Diaion HP-20 resin (DIR); (c and d) biocatalysts (APS-AC-CBS, APS-AC-FCBS, and APS-DIR), and Amano lipase (APS).

Region II (150 to 500 °C) (Figure 6a) corresponds to the decomposition of organic matter, cellulose, and hemicellulose. Weight loss in this region was approximately 40% for CBS and FCBS and 8% for DIR, while the AC (AC-CBS and AC-FCBS) remained thermally stable. This confirms that all organic compounds were fully carbonized during the carbonization process. As shown in Figure 6b, endothermic peaks appear in the same temperature range, with FCBS exhibiting a lower combustion rate than CBS, reinforcing the effectiveness of the pretreatment in degrading lignocellulosic compounds.

Studies have investigated the effect of alkaline extraction on lignocellulosic degradation, yielding satisfactory results.^62^ The literature demonstrated that hemicellulose, due to its predominantly amorphous structure, decomposes first, within the 220–310 °C range. Conversely, cellulose, which is more crystalline, decomposes between 310 and 360 °C. These distinctions are clearly reflected in the thermal analysis of the materials.

Region II (Figure 6c) also provides insights into the thermal stability of the enzyme and biocatalysts. The free lipase (APS) exhibited a 70% mass loss, which was significantly reduced after immobilization. The biocatalysts APS-DIR, APS-AC-FCBS, and APS-AC-CBS exhibited gradual mass losses of 47%, 20%, and 12%, respectively. Part of this weight loss is attributed to the removal of water molecules strongly bound to the carbon matrix an interaction absent in the synthetic support as well as the decomposition of organic amino groups.^63^

Comparing the commercial biocatalyst (APS-DIR) with the fermented biomass-based biocatalyst (APS-AC-FCBS), the latter exhibited a 27% lower mass loss compared to APS-AC-CBS. This suggests greater thermal stability, likely due to stronger interactions between the carbon matrix and organic components (lipase). Previous studies have linked lower mass loss in immobilized lipase to increased thermal stability, attributed to enzyme-support interactions.^64^ The DTG curve further supports this observation, revealing a pronounced endothermic peak indicative of a higher degradation rate in the free enzyme. In contrast, degradation in the biocatalysts occurred more gradually.

In region III (500 to 1000 °C) (Figure 6a), continuous mass loss was observed, mirroring events described in region II. Mass loss was 95% for CBS and 90% for FCBS, with the fermented biomass exhibiting 5% lower mass loss. This reduction is likely due to the degradation of lignocellulosic agri-food byproducts, particularly lignin, a highly recalcitrant component. As a result, FCBS decomposed more readily at lower temperatures, whereas CBS retained these compounds, leading to more extensive mass loss. Additionally, AC-FCBS demonstrated greater thermal stability, likely due to its higher fixed carbon content, which enhances the structural integrity of the support and broadens its applicability over a wide temperature range.^65^ The DTG curve in Figure 6b shows endothermic peaks that corroborate these findings.

In region III (500 to 1000 °C) (Figure 6c), the APS-AC-CBS biocatalyst exhibited approximately 13% greater mass loss than APS-AC-FCBS. This suggests that APS-AC-FCBS possesses greater thermal stability, likely due to the SSF process applied to the precursor material, which enhanced its absorptive properties.^14^ In contrast, APS-DIR and APS continued the degradation trends observed in region II, indicating lower stability. While the APS was almost entirely degraded in region II, immobilization significantly improved its thermal stability—an essential factor for industrial applications.

#### Application of Biocatalysts in Aroma Compound Production

The biocatalysts (APS-AC-CBS and APS-AC-FCBS) demonstrated maximum yields of 75% and 81%, respectively (Figure 7). Compared to the synthetic support (APS-DIR), esterification yields were found to be similar. Although the synthetic support exhibited yields 10% higher than APS-AC-FCBS and 15% higher than APS-AC-CBS, its industrial application is costly due to its derivation from nonrenewable sources and high production costs. According to Sigma-Aldrich, 1 kg of this resin costs an average of $410.00.

**Figure 7:**
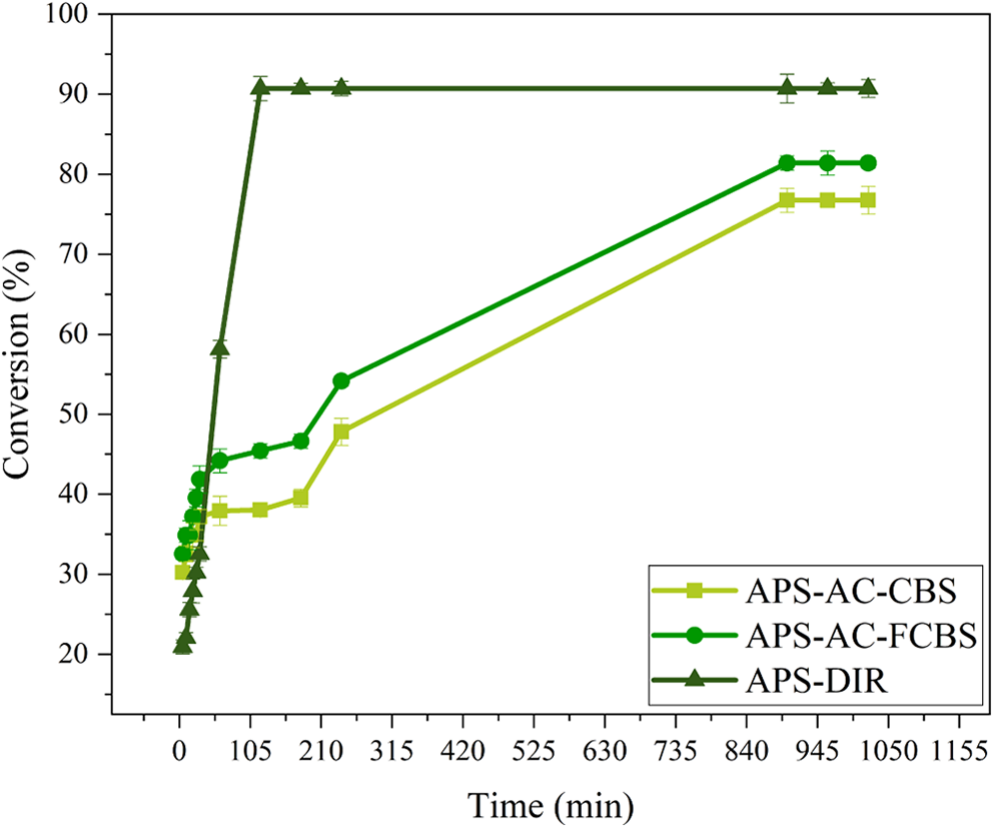
Residual esterification yield (%) of the biocatalysts obtained by different immobilization strategies: APS-AC-CBS (lipase immobilized on activated carbon from cocoa bean shell), APS-AC-FCBS (lipase immobilized on activated carbon from fermented cocoa bean shell), and APS-DIR (lipase directly immobilized on the support). Results are shown as a function of reaction time, with data expressed as mean ± standard deviation from duplicate experiments.

In contrast, the biocatalyst produced from fermented biomass (APS-AC-FCBS) represents a sustainable approach by valorizing previously underutilized agri-food byproduct, while achieving comparable results. Moreover, the use of fermented biomass in AC production supports the circular economy by completing the carbon cycle and helping to mitigate air and water pollution.^33^

A study conducted in 2021 evaluated a synthetic support immobilized with 40 mg of *Candida rugosa* lipase for the synthesis of the aroma ester hexyl butyrate, achieving a maximum conversion yield of 89.1% in 150 min.^18^ Similarly, in 2020, researchers investigated the use of AC derived from lignocellulosic agri-food byproducts for lipase immobilization, applying the biocatalyst in the synthesis of isoamyl acetate ester and obtaining conversion rates of approximately 93% within 180 min.^13^

While these studies yielded relevant results, the present work differs by employing lower enzyme concentrations in combination with renewable and economically viable support. Additionally, the developed biocatalyst demonstrated competitive performance in both conversion efficiency and reaction time, underscoring its effectiveness.

A key contribution of this study is the enhanced bioprocessing of the AC support, which improved enzyme-support interactions and optimized catalytic properties. This innovative approach represents a significant advancement over previous methodologies, offering a more sustainable and high-performance alternative. Recent studies reinforce the importance of exploring lignocellulosic materials as effective supports for enzyme immobilization, emphasizing their versatility and sustainability.^16,47^ Consequently, this study not only complements the existing literature but also demonstrates substantial improvements in catalytic efficiency, cost-effectiveness, and environmental impact.

From a commercial perspective, the findings of this study are highly promising, given the reduced enzyme concentrations required and the simple yet efficient immobilization method.^66^ Previous research has reported the application of immobilized lipase on nonrenewable synthetic supports, which are associated with high operational costs. For instance, a biocatalyst using 40 mg of enzyme was reused for up to seven cycles, achieving conversions of up to 89% in the synthesis of hexyl butyrate ester.^18^

In contrast, the APS-AC-FCBS developed in this study achieved an 81% conversion rate in the synthesis of the same ester using only 3 mg of lipase, while also allowing reuse for up to ten cycles. This efficiency is attributed to the structural properties of the lignocellulosic-derived AC support, which provides a larger surface area and enhanced porosity due to the bioprocessing approach.

These results highlight the commercial viability of the developed biocatalysts, particularly for the production of high-value aroma compounds. The reduced enzyme consumption and extended reuse cycles offer significant economic and environmental benefits, making the process more sustainable. Therefore, the biocatalyst proposed in this study represents an innovative and efficient alternative with the potential to surpass recent biocatalysts described in the literature for aroma ester synthesis.^54,67^

#### Stability Tests of the Biocatalysts

The reuse of immobilized catalysts is a crucial factor that directly impacts the economic feasibility of their application, particularly in large-scale industrial sectors such as the food and pharmaceutical industries. Recent studies have shown that the efficient reuse of biocatalysts significantly reduces operational costs by minimizing the need for frequent enzyme replacement, which is one of the most expensive components of the process.^17^

In this context, the recyclability and recovery of immobilized APS catalysts (APS-AC-CBS, APS-AC-FCBS) and APS-DIR were systematically evaluated through repeated-use cycles, as illustrated in Figure 8. By the seventh cycle, a 30% decrease in the conversion capacity of APS-DIR was observed. However, APS-AC-FCBS remained remarkably stable, showing only a ∼5% reduction, indicating a 25% higher operational stability compared to the synthetic biocatalyst (APS-DIR).

**Figure 8:**
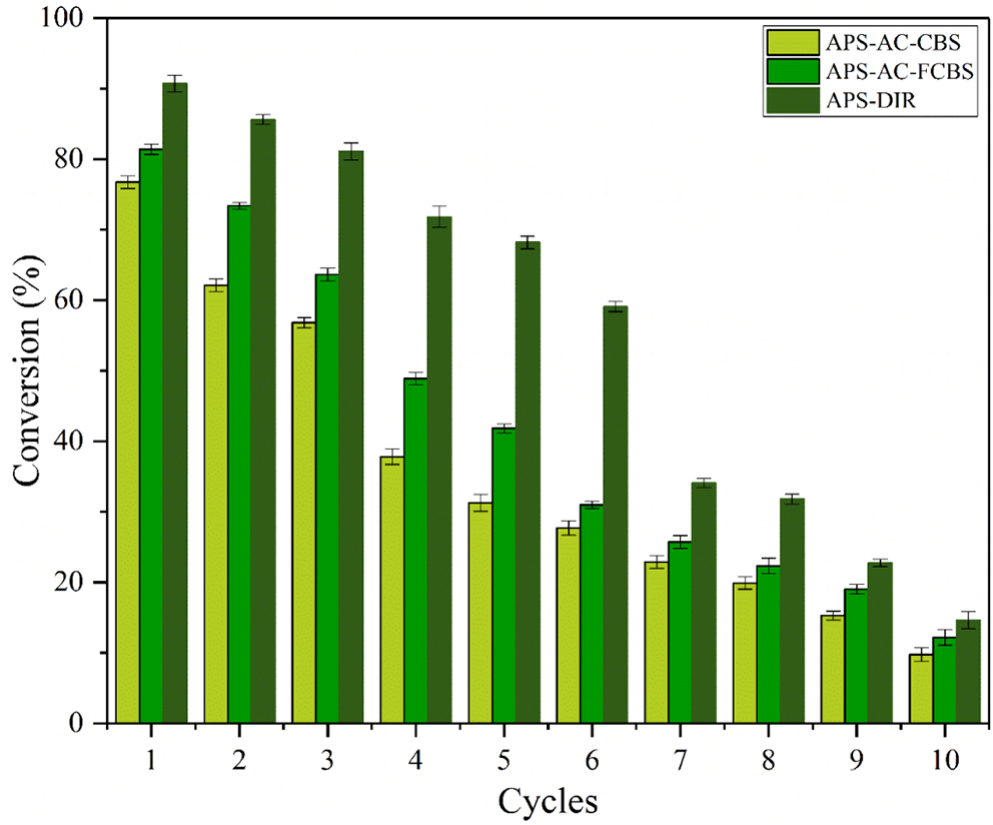
Reuse cycle of the biocatalysts (APS-AC-CBS, APS-AC-FCBS, and APS-DIR) for the conversion of butyric acid and hexanol in the esterification reaction of hexyl butyrate.

The results further demonstrate that APS-AC-FCBS, APS-AC-CBS, and APS-DIR can be recovered from the reaction medium via simple filtration followed by washing with cold hexane, and successfully reused for at least ten consecutive cycles, maintaining conversion efficiencies of approximately 18%, 12%, and 20%, respectively, relative to their initial activity. By the tenth cycle, APS-AC-FCBS and APS-DIR exhibited nearly equivalent conversion capacities, suggesting that APS-AC-FCBS provides robust performance and can effectively replace synthetic biocatalysts while enabling efficient enzyme recovery and sustained activity. This enhanced recyclability is attributed to the strong adsorption of lipase onto the surface of AC-FCBS, which minimizes enzyme desorption over multiple cycles, combined with the improved physicochemical properties of AC-FCBS, such as increased surface area, porosity, and the presence of hydrophobic functional groups, all of which enhance enzyme–substrate interactions and catalytic stability.^69^

Esterification reactions were carried out in heptane, a hydrophobic solvent commonly applied in lipase-catalyzed processes for preserving enzymatic activity.^68^ The hydrophobic nature of AC-FCBS, along with its enhanced porosity and surface area, contributed decisively to maintaining enzyme activity during repeated cycles, further supporting the mechanistic explanation of its superior performance.^70^

Moreover, the superior catalytic efficiency and stability of APS-AC-FCBS are reinforced by the interfacial activation of lipase, where the hydrophobic support surface and the use of heptane facilitate conformational changes that expose active sites, enhancing activity and thermal stability over multiple uses.

Therefore, this study clearly demonstrates that the reuse and recovery of biocatalysts derived from fermented biomass not only maintain high catalytic efficiency comparable to synthetic supports but also provide significant economic and environmental advantages, reinforcing the potential of low-cost, renewable supports in industrial applications.

#### Gas Chromatography GC-FID

The GC-FID was employed to identify and confirm the synthesis of hexyl butyrate by direct comparison between the standard ester and the synthesized product.

The analysis of the reference compound (hexyl butyrate standard–Figure 9a) showed a well-defined and characteristic chromatographic peak at a retention time of 5.7 min, establishing the identification parameter for the desired product, hexyl butyrate (CH_3_(CH_2_)_2_COO­(CH_2_)_5_CH_2_).

**Figure 9:**
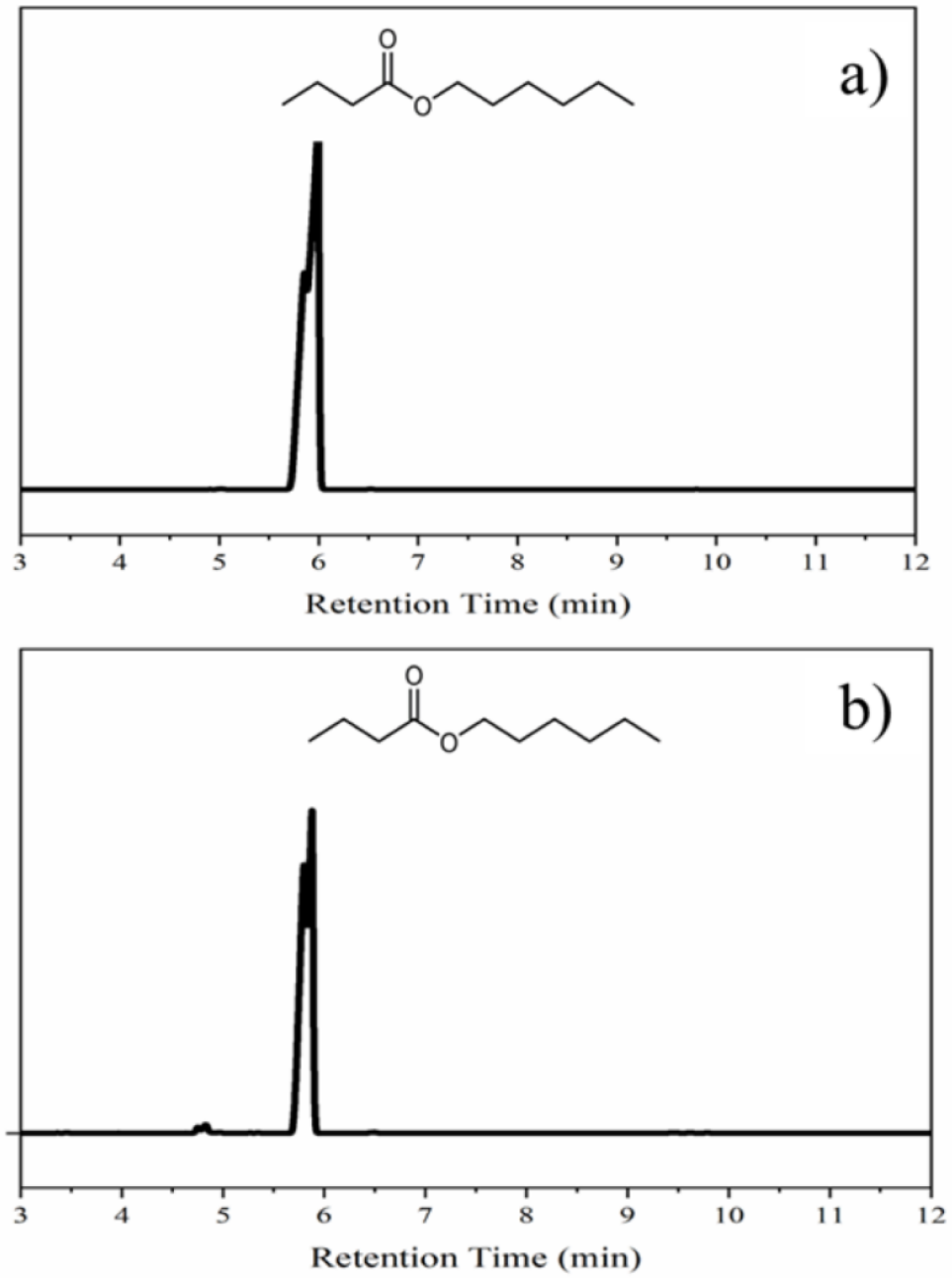
GC-FID analysis chromatograms of hexyl butyrate (a) commercial standard; (b) synthesized product obtained after the biocatalyst-catalyzed esterification reaction.

Subsequently, the reaction mixture obtained from the biocatalyst-mediated synthesis was analyzed by GC (Figure 9b). The chromatogram of the synthesized product displayed a prominent main peak eluted at exactly the same retention time (5.7 min) observed for the hexyl butyrate standard.

The coincidence of retention times unequivocally confirms that the synthesized compound corresponds to hexyl butyrate. Furthermore, the high peak intensity and the absence of significant secondary peaks in Figure 9b provide strong visual evidence of the selective formation of the ester and the high catalytic efficiency of the system in the esterification reaction.

## Conclusion

This study highlights the potential of activated carbons derived CBS as innovative and sustainable materials for lipase immobilization and aroma ester production. The CBS, rich in lignin, cellulose, and hemicellulose, has proven to be an excellent raw material for AC production, with the lignocellulosic compounds being pivotal in pore formation during carbonization. Bioprocessing with *Aspergillus niger* not only increased the surface area of the activated carbons but also improved the material’s efficiency, as evidenced by AC-FCBS, which exhibited a 20% higher yield compared to AC-CBS.

SSF of the lignocellulosic biomass before carbonization contributed to the reduction of lignin recalcitrance, allowing for efficient carbonization at lower temperatures, resulting in reduced operational costs. The biotransformation process endowed the activated carbons with superior characteristics, including high adsorption capacity, making them promising for applications as enzyme supports under the conditions tested.

Lipase immobilization on the activated carbons, particularly on AC-FCBS, showed comparable performance to synthetic supports, but with the advantage of being low-cost and derived from renewable materials. The high porosity and surface area of the bioprocessed carbons ensured effective enzyme-support interactions, minimizing saturation and blocking effects, and maximizing catalytic efficiency even at high enzyme concentrations.

The reuse of the biocatalysts demonstrated stability, with APS-AC-FCBS retaining 95% of its efficiency after 10 cycles of use, compared to a 30% decline observed in synthetic supports. These results support the potential economic and environmental benefits of using renewable biocatalytic supports within the scope of this study, offering an alternative to synthetic materials under the tested experimental conditions.

The development of biocatalysts based on AC derived from organic waste not only provides a solution for waste valorization but also may contribute to more ecological and economically viable industrial processes, while further studies are needed to confirm broad industrial applicability, aligning with the principles of the circular economy.

## Abbreviations

AC: Activated carbon
APS: Amano Lipase PS
CBS: Cocoa Bean Shells
FCBS: Fermented Cocoa Bean Shells
AC-CBS: Activated carbon derived from cocoa bean shells
AC-FCBS: Activated carbon derived from fermented cocoa bean shells
DIR: Diaion HP-20 resin
APS-AC-CBS: Amano Lipase PS immobilized on Activated carbon derived from cocoa bean shells
APS-AC-FCBS: Amano Lipase PS immobilized on Activated carbon derived from fermented cocoa bean shells
APS-DIR: Amano Lipase PS immobilized on Diaion HP-20 resin
SSF: Solid-state fermentation
S-APS: Amano Lipase PS enzyme solution
S-AC-CBS: Supernatant after Amano Lipase PS immobilization on activated carbon derived from cocoa bean shells
S-AC-FCBS: Supernatant after Amano Lipase PS immobilization on activated carbon derived from fermented cocoa bean shells
S-DIR: Supernatant after Amano Lipase PS immobilization on Diaion HP-20
